# Unveiling the link: Screen dependency and sleep pattern disturbances in preadolescent children

**DOI:** 10.6026/973206300213546

**Published:** 2025-10-31

**Authors:** Devikrishna R, Madhavi S, Lilly Christopher, Buvaneswari Ramakrishnan, Jamunarani P, Sivasubramanian N, Mahalakshmi B, Ponmari K

**Affiliations:** 1 Department of Psychiatric Nursing, Meenakshi academy of higher education and research, Chennai, Tamilnadu, India; 2 Department of Medical & Surgical Nursing, KMCH College of Nursing, Coimbatore, The Tamilnadu DR. M.G.R Medical University, Chennai, India; 3 Department of Medical & Surgical Nursing, Meenakshi Academy of Higher Education and Research, Chennai, Tamilnadu, India; 4 Department of Psychiatric Nursing, KMCH College of Nursing, Coimbatore, The Tamilnadu DR. M.G.R Medical University, Chennai, India; 5 Department of Psychiatric Nursing, Nootan College of Nursing, Sankalchand Patel University, Visnagar, Gujarat - 384315, India; 6 Department of Paediatric Nursing, Nootan College of Nursing, Sankalchand Patel University, Visnagar, Gujarat - 384315, India; 7 Department of Medical & Surgical Nursing, SGRRIM & HS College of Nursing, Shri Gururam Rai University, Dehradun, Uttarakhand - 248001, India

**Keywords:** Screen dependency, sleep pattern disturbance, preadolescent children, sleep quality

## Abstract

Sleep pattern disturbances among 75 preadolescent children (aged 9-12 years) at The NGP School, Coimbatore, in 2025, with 42 identified as having screen dependency is of interest. Using the Digital Addiction Scale for Children (DASC) and Sleep Quality Scale (SQS), data were collected via structured interviews. Using the SQS, sleep disturbances were categorized as mild (31%, 1-43), moderate (33%, 44-87) and severe (36%, 88-130). Significant associations were found between age and sleep disturbances (χ^2^=27.06, df=4, p<0.05) and religion with screen dependency (χ^2^=18.96, df=4, p<0.05). Thus, we show the need for interventions to address screen-related sleep issues in preadolescents.

## Background:

Preadolescence, spanning ages 9-12, is a critical developmental stage where children begin to assert independence and engage more with digital devices [1]. Screen dependency, encompassing compulsive use of smartphones, tablets, computers and other digital devices despite adverse effects, is a growing concern among preadolescents [2]. Excessive screen time is associated with sleep pattern disturbances, including insufficient sleep duration, delayed bedtimes and poor sleep quality, which can impair cognitive, emotional and social development [3]. Globally, the prevalence of screen dependency in preadolescents ranges from 10% to 67% [4], with India reporting 6% to 49% [6]. In the US, studies indicate a 40.9% prevalence of screen dependency, with a moderate positive correlation (r=0.368, p=0.001) between screen use and sleep disturbances [5]. Research consistently links screen time to sleep issues. Beena *et al.* (2023) found that 34.3% of children aged 6-12 in South Kerala slept less than the recommended 9 hours due to pre-bedtime screen exposure, increasing bedtime resistance and sleep disorders [7]. Similarly, Li *et al.* reported that Chinese school children experienced sleep disturbances linked to media use [8]. Another study by Qanash *et al.* (2021) found that 71.9% of Saudi adolescents had poor sleep quality due to smartphone addiction [9]. Recent reviews emphasize that early digital exposure before the age of 10 may disrupt melatonin secretion and circadian rhythm, predisposing children to chronic sleep deprivation [10]. Furthermore, emerging evidence highlights that family screen habits and lack of parental monitoring significantly contribute to unhealthy screen use and poor sleep hygiene among preadolescents [11]. Therefore, it is of interest to assess the level of screen dependency and sleep pattern disturbances among preadolescents in Coimbatore, India, hypothesizing that higher levels of screen dependency are significantly associated with increased sleep disturbances.

## Methodology:

## Study design and setting:

A descriptive cross-sectional design was employed at The NGP School, Coimbatore, from January 10-16, 2025, targeting preadolescents aged 9-12 years with screen dependency.

## Population and sampling:

The accessible population included preadolescents at The NGP School. A sample of 75 children was selected using simple random sampling, including both genders able to communicate in English or Tamil, excluding those with mental challenges or hearing loss.

## Data collection:

Data were collected after obtaining permissions from KMCH College of Nursing and The NGP School. Structured interviews, averaging 20 minutes per participant, used the Digital Addiction Scale for Children (DASC; scores 0-100, higher scores indicating greater dependency) and the Sleep Quality Scale (SQS; scores 0-130, categorized as mild [1-43], moderate [44-87] and severe [88-130] disturbances).

## Data analysis:

Descriptive statistics (frequency, percentage) analyzed demographic characteristics, screen dependency and sleep disturbances. Chi-square tests assessed associations between sleep disturbances, screen dependency and demographic variables (p<0.05).

## Ethical considerations:

Informed consent was obtained from school authorities and parents, ensuring participant confidentiality and voluntary participation.

## Results:

Table 1 (see PDF) most preadolescents (51%) were aged 11-12 years, and a nearly equal gender distribution was observed (51% male and 49% female). A majority (87%) belonged to the Hindu religion. Over half of the parents were graduates (53%), and 47% were postgraduates. Regarding employment, 59% were self-employed and 41% worked full-time. More than half (53%) belonged to nuclear families, while 38% lived in joint families. All parents were married. Most families (84%) reported a monthly income above ₹50,000, indicating a predominantly upper-middle socioeconomic background. Table 2 (see PDF) shows that a significant association of the study variable with age (χ^2^ = 27.06, p < 0.05) and religion (χ^2^ = 18.96, p < 0.05). Other demographic factors such as gender, parental education, occupation, type of family, marital status, and monthly income showed no significant association. Figure 1 (see PDF) is a pie chart showing mild (31%), moderate (33%) and severe (36%) sleep disturbances among the 42 preadolescents with screen dependency.

## Discussion:

This study confirms a significant association between screen dependency and sleep pattern disturbances among preadolescents, with 33% exhibiting moderate disturbances and 36% severe disturbances, highlighting the urgent need for intervention. These findings align with Beena *et al.* (2023), who reported that 34.3% of children aged 6-12 in South Kerala slept less than the recommended 9 hours due to pre-bedtime screen exposure, leading to increased bedtime resistance and sleep disorders [7]. Shenghili and Jiang (2023) found that 48.8% of Chinese schoolchildren experienced sleep disturbances linked to media use [8], while Qanash *et al.* (2021) reported that 71.9% of Saudi adolescents had poor sleep quality due to smartphone addiction [9]. The significant association between age and sleep disturbances (χ^2^=27.06, p<0.05) indicates that older preadolescents (11-12 years) are more vulnerable, possibly due to increased autonomy in device use. The association between religion and screen dependency (χ^2^=18.96, p<0.05) may reflect cultural differences in device access or parenting practices among Hindu and Christian families in Coimbatore, warranting further exploration. This aligns with Hysing (2015), who found that preadolescents using screens for over four hours daily slept 60-90 minutes less, with mobile phones having the most significant impact [20]. Levenson *et al.* (2017) noted that social media use within 30 minutes of bedtime independently predicts sleep disturbances [12]. Trott *et al.* (2022) found that 87% of studies linked screen use to poor sleeps outcomes, particularly with social networking and gaming [13].

The physiological mechanisms involve blue light from screens suppressing melatonin production, delaying sleep onset and reducing sleep quality [14]. This is consistent with Mayura *et al.* (2022), who found that 15.6% of males and 23.5% of females in India had sleep problems associated with smartphone use [15]. This study confirms that older preadolescents with greater autonomy in device use are more vulnerable to sleep disturbances, consistent with findings showing bedtime screen behaviors predict shorter sleep duration [16]. A systematic review found 90% of studies reported negative sleep outcomes linked to screen time, especially when used before bedtime [1]. Specific screen activities such as computer use and gaming are associated with shorter sleep and more daytime sleepiness [17], while screen time over four hours daily correlates with higher depression and anxiety [18]. An intervention reducing screen use after 9 PM resulted in improved sleep duration and daytime vigilance [19], highlighting evening screen exposure as a key modifiable target. Limitations include potential recall bias in self-reported screen use and sleep patterns, the cross-sectional design limiting causal inferences and confinement to a single school, reducing generalizability. Future research should explore longitudinal effects, intervention efficacy and the role of comorbidities like anxiety or ADHD.
